# Impact of Different KDIGO Criteria on Clinical Outcomes for Early Identification of Acute Kidney Injury after Non-Cardiac Surgery

**DOI:** 10.3390/jcm11195589

**Published:** 2022-09-23

**Authors:** Jingwen Fu, Junko Kosaka, Hiroshi Morimatsu

**Affiliations:** Department of Anesthesiology and Resuscitology, Okayama University Graduate School of Medicine, Dentistry and Pharmaceutical Sciences, 2-5-1, Shikata-cho, Kita-ku, Okayama 700-8558, Japan

**Keywords:** acute kidney injury, KDIGO definition, serum creatine, urine output, early identification

## Abstract

The Kidney Disease Improving Global Outcomes (KDIGO) guidelines are currently used in acute kidney injury (AKI) diagnosis and include both serum creatinine (SCR) and urine output (UO) criteria. Currently, many AKI-related studies have inconsistently defined AKI, which possibly affects the comparison of their results. Therefore, we hypothesized that the different criteria in the KDIGO guidelines vary in measuring the incidence of AKI and its association with clinical outcomes. We retrospectively analyzed that data of patients admitted to the intensive care unit after non-cardiac surgery in 2019. Three different criteria used to define AKI were included: UOmean, mean UO < 0.5 mL/kg/h over time; UOcont, hourly UO < 0.5 mL/kg/h over time; or SCR, KDIGO guidelines SCR criteria. A total of 777 patients were included, and the incidence of UOmean-AKI was 33.1%, the incidence of UOcont-AKI was 7.9%, and the incidence of SCR-AKI was 2.0%. There were differences in the length of ICU stay and hospital stay between AKI and non-AKI patients under different criteria. We found differences in the incidence and clinical outcomes of AKI after non-cardiac surgery when using different KDIGO criteria.

## 1. Introduction

Acute kidney injury (AKI) is a serious complication in the perioperative period [1]. Perioperative AKI is associated with increases in patient mortality, length of hospital stays, and medical costs [2]. Many perioperative conditions, such as intravascular insufficiency of effective blood volume and organ perfusion, nephrotoxic drug use, and contrast exposure, may contribute to the development of AKI [3]. Therefore, early recognition of and intervention for AKI in the perioperative period play crucial roles in maintaining patient safety.

The first diagnostic criteria for AKI proposed by the Acute Dialysis Quality Initiative group in 2004 were risk, injury, failure, loss, and end-stage renal disease (RIFLE), which included the serum creatinine (SCR) and urine output (UO) criteria [4]. In two subsequent updates of the Acute Kidney Injury Network (AKIN) and Kidney Disease Improving Global Outcomes (KDIGO) criteria, the SCR criteria were changed twice, whereas the UO criteria were continued as defined in RIFLE [5,6]. The current KDIGO definition is recommended for the assessment of AKI, and it defines the UO criteria for AKI as UO < 0.5 mL/kg/h for 6 h.

However, we found discrepancies in the diagnostic criteria for AKI in the recent clinical studies. Many studies have used only SCR criteria as diagnostic criteria for AKI and ignored the UO criteria [7,8,9,10,11,12]. In some AKI studies that used the UO criteria, the description of the UO criteria was unclear [13,14,15]; it was also unclear whether the average UO or the hourly UO over a certain period met the criteria as the basis for diagnosis. The KDIGO guidelines reported that the specific usage of the UO criteria is unclear and that further evaluation of the significance of the UO criteria for the staging diagnosis of AKI is needed [6]. The inconsistent use of these criteria in the recent AKI-related clinical studies makes it difficult to compare their results. Therefore, we believe that it is necessary to use consistent criteria for AKI diagnosis.

We aimed to assess whether the incidence and staging of AKI in patients admitted to the intensive care unit (ICU) after non-cardiac surgery is the same when different criteria are used for the early identification of postoperative AKI. In addition, we compared the association between patients with AKI and clinical outcomes among the different criteria.

## 2. Materials and Methods

### 2.1. Study Design

In this single-center retrospective observational study, we selected patients who underwent non-cardiac surgery for more than 2 h and were admitted to the ICU postoperatively at Okayama University Hospital between 1 January 2019 and 31 December 2019 to participate in the study. The Okayama University Medical Ethics Committee approved the study protocol.

#### 2.1.1. Inclusion Criteria

Patients included were aged ≥20 years, underwent non-cardiac surgery for more than 2 h at Okayama University Hospital, and were admitted to the ICU for at least one night postoperatively. In our university hospital, patients are admitted to the ICU for postoperative follow-up, depending on the type of surgery (e.g., cardiac, respiratory, esophageal, hepatobiliary, neurosurgical, head and neck surgery) and/or their co-morbidities.

#### 2.1.2. Exclusion Criteria

We excluded patients who underwent urological, neurological, or obstetrical surgery; those who underwent transplantation; those in whom intraoperative contrast agent was used; patients with end-stage renal disease; and those requiring preoperative maintenance dialysis. If a patient underwent multiple surgeries during the study period, only data from the first surgery and ICU admission were selected. Patients with missing essential data were also excluded from the study.

### 2.2. Data Collection

Data were extracted from an electronic medical record system. We collected data on basic patient characteristics (sex, age, body mass index (BMI), length of ICU stay, and length of hospital stay), comorbidities (hypertension, diabetes, history of cancer, and chronic kidney disease), preoperative laboratory results, surgical information (department, elective or emergency, and open or laparoscopic surgery), and anesthesia information (American Society of Anesthesiologists classification (ASA-PS), total intravenous anesthesia (TIVA) or inhalation, and anesthesia duration). Patient survival at 90 days and 1 year postoperatively was obtained using the electronic medical record system. The body weight used to calculate the UO was derived from the data recorded at ICU admission. We collected SCR data within 6 months preoperatively and 7 days postoperatively. For urine output collection, if the patient was in the ICU for more than 48 h, the hourly urine output was collected for the first 48 h after the patient was admitted to the ICU. If the patient was in the ICU for less than 48 h, all hourly urine output before the patient was discharged from the ICU was collected. We obtained hourly UO data from the ICU medical record system, and if there was an hour of unrecorded UO, then an average from the next hour’s UO was used.

### 2.3. Definition

#### 2.3.1. AKI Identification and Staging

The identification and staging of AKI in our study were based on the KDIGO clinical practice guidelines. The KDIGO guidelines define ungraded AKI as an increase in SCR ≥ 0.3 mg/dl (≥26.5 µmol/L) within 48 h or a known or presumed increase in SCR to ≥1.5 times the baseline within 7 days; or UO < 0.5 mL/kg/h for 6 h [6]. 

AKI stage 1 is defined in the KDIGO guidelines as SCR reaching 1.5–1.9 times the baseline or an increase of ≥0.3 mg/dL (>26.5 µmol/L); or UO < 0.5 mL/kg/h for 6–12 h. AKI stage 2 is SCR reaching 2.0–2.9 times the baseline; or UO < 0.5 mL/kg/h for ≥12 h. AKI stage 3 is defined as an SCR of up to 3.0 times the baseline or SCR increased to ≥4.0 mg/dl (353.6 µmol/L) or initiation of renal replacement therapy; or UO < 0.3 mL/kg/h for ≥24 h or anuria for ≥12 h. In this study, we defined early identification as the identification of AKI within 48 h of postoperative admission to the ICU.

#### 2.3.2. Baseline Definition of SCR

We collected all SCR from patients within 6 months prior to surgery and selected the lowest of these values as the baseline. 

#### 2.3.3. Definition of Different UO Criteria

The KDIGO guidelines define the UO criteria as UO < 0.5 mL/kg/h or <0.3 mL/kg/h for 6, 12, or 24 h. We shifted the hourly urine output values obtained within 48 h of the patient’s admission to the ICU in fixed time periods of 6, 12, or 24 h at 1-hour intervals. Based on a previous study, we proposed two methods for the calculation of UO criteria in our study [16].

- UOmean: Mean UO < 0.5 mL/kg/h or < 0.3 mL/kg/h at 6, 12, or 24 h.
- UOcont: UO < 0.5 mL/kg/h or <0.3 mL/kg/h per hour continuously for 6, 12, or 24 h.

### 2.4. Outcomes

Our primary outcome was the incidence of AKI identified using the following three different criteria within 48 h of admission to the ICU after non-cardiac surgery: UOmean, UOcont, and SCR.

Secondary outcomes were differences in the length of ICU stay, the length of hospital stay, 90-day mortality, and 1-year mortality between AKI and non-AKI patients with different criteria.

### 2.5. Statistical Analysis

For representation of variables, continuous variables were tested for obedience to normal distribution using the Shapiro–Wilk test. Normally distributed variables are expressed as mean ± standard deviation and were compared using the t-test; abnormally distributed variables are expressed as median (Q1–Q3) and were compared using the Mann–Whitney U test. Categorical variables are expressed as numbers (proportions) and were compared using the chi-square test. For the analysis of clinical outcomes, we further used the cox proportional risk model to assess the association between patients with AKI and 90-day and 1-year mortality under different criteria. The unadjusted hazard ratios for AKI patients with 90-day and 1-year mortality under different criteria were first obtained; these were then adjusted using the model, and finally the adjusted hazard ratios were obtained. Regarding the composition of the model, factors within the baseline characteristics of the all patients were first subjected for univariate logistic regression with 1-year mortality (Supplementary Materials Table S1). Based on the results in Supplementary Materials Table S1, we selected the following 7 factors as our adjusted models to obtain adjusted hazard ratios: sex, BMI, preoperative hemoglobin, preoperative albumin, emergency surgery, TIVA, and anesthesia duration. Statistical differences were considered statistically significant at a *p*-value of <0.05. Statistical analyses were performed using STATA/SE version 17.0 (StataCorp, College Station, TX, USA).

## 3. Results

### 3.1. Patient

**Figure 1:**
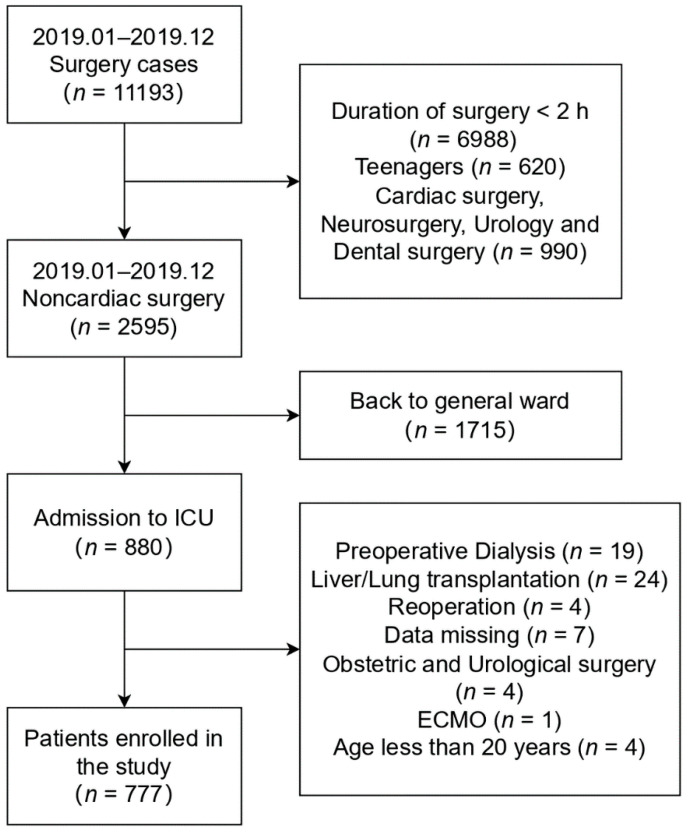
Flow chart. ICU = intensive care unit; ECMO = extracorporeal membrane oxygenation.

During the study period, we obtained data from 880 patients admitted to the ICU after non-cardiac surgery. Of these, 103 were excluded due to preoperative dialysis (*n* = 19), liver or lung transplantation (*n* = 24), reoperation (*n* = 44), essential data loss (*n* = 7), and other reasons (*n* = 9). The final study population included 777 patients (Figure 1). The demographic data and clinical characteristics of all patients enrolled in the study are shown in Table 1; 284 patients were women (36.6%), median age was 68.8 years (interquartile range (IQR) 57.0–75.0 years), median preoperative creatinine was 0.78 mg/dL (IQR 0.64–0.93 mg/dL), and 338 patients (43.5%) underwent abdominal surgery, which accounted for the highest percentage of non-cardiac surgeries. Eighty patients (10.3%) underwent emergency surgery, 361 (46.5%) underwent laparoscopic surgery, 163 (20.9%) were classified as grade III or IV according to ASA-PS, and 283 (36.4%) underwent TIVA. The median anesthesia duration was 6.3 h (IQR 4.6–9.0 h). Supplementary Materials Table S2 shows the baseline characteristics of patients identified with AKI and non-AKI under different criteria.

### 3.2. Incidence

**Figure 2:**
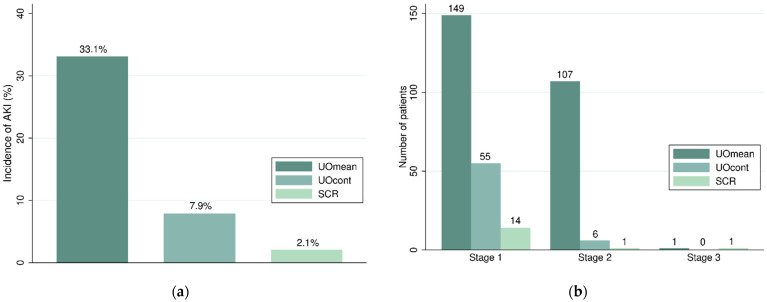
(**a**) Incidence of AKI identified by different criteria within 48 h of admission to the ICU after non-cardiac surgery. (**b**) Incidence of each stage of AKI. UOmean is mean urine output <0.5 mL/kg/h or <0.3 mL/kg/h within a sliding window of 6, 12, or 24 h. UOcont is urine output <0.5 mL/kg/h or <0.3 mL/kg/h hourly within a sliding window of 6, 12, or 24 h. SCR is serum creatinine increase of ≥0.3 mg/dL or 1.5–3 times the baseline within 48 h.

The incidence of AKI within 48 h varied significantly depending on the AKI criteria used (Figure 2a). The incidence of UOmean-AKI was 33.1%, the incidence of UOcont-AKI was 7.9%, and the incidence of SCR-AKI was 2.0%. Thus, the incidence of UOmean-AKI was approximately 16 times higher than that of SCR-AKI. 

### 3.3. AKI Severity

The patients were stratified according to AKI severity among the different criteria, and the ability to identify each stage of AKI varied considerably between the criteria (Figure 2b). Under UOmean and UOcont, more stage 1 AKI cases were identified than with SCR (149 patients in UOmean, 55 in UOcont, and 14 in SCR). Moreover, UOmean showed threefold increase in stage 1 AKI identification than that in UOcont. Regarding the incidence of stage 2 AKI, AKI identified by UOmean was much higher than that by UOcont and SCR, and the incidence of stage 2 AKI was low for UOcont and SCR (107 in UOmean, 6 in UOcont, and 1 in SCR). In all criteria, there were only a small number of patients with stage 3 AKI (1 in UOmean and SCR and none in UOcont). SCR could detect only a small number of patients in any stage of AKI; however, UOmean could detect many patients with AKI, especially in the early stages.

### 3.4. Clinical Outcomes

Table 2 demonstrates the association between AKI and clinical outcomes under different criteria. Regarding the length of ICU stay and hospital stay, there were differences in the length of ICU stay and hospital stay for AKI and non-AKI under the three criteria. The length of ICU stay and hospital stay were longer in SCR-AKI than in UOmean-AKI and UOcont-AKI.

Regarding the 90-day and 1-year mortality results, our results were different between UOmean-AKI and non-AKI patients in 90-day and 1-year mortality. The adjusted hazard ratios of UOmean-AKI for 90-day and 1-year mortality were obtained using a cox proportional hazards model and adjusting for covariates (sex, BMI, preoperative hemoglobin, preoperative albumin, emergency surgery, TIVA, and anesthesia duration). The adjusted hazard ratio of UOmean-AKI for 90-day mortality was 5.72 (1.15–28.59); for 1-year mortality this was 1.72 (0.89–3.30). The results of hazard ratios for 90-day and 1-year mortality for UOcont-AKI and SCR-AKI are presented in Supplementary Materials Table S3.

## 4. Discussion

In the present study, we selected different criteria based on the KDIGO definition for the early identification of AKI after non-cardiac surgery to examine whether there are differences in the incidence and degree of association with clinical outcomes. We found that the different criteria resulted in different incidences, with UOmean-AKI having the highest incidence of 33.1%; this was approximately 16 times higher than the incidence of SCR-AKI (2.0%), which was the lowest. There were differences in the length of ICU stay and hospital stay for AKI and non-AKI patients identified under each criterion. These findings are consistent with our hypothesis that there are differences in the incidence of AKI and clinical outcomes under each criterion using different criteria to early identification of AKI.

When we used different criteria to identify AKI, we found that the UO criteria indicated higher incidence of AKI than the SCR criteria. Other studies have confirmed that the use of only UO criteria or the addition of UO criteria to SCR criteria in identifying AKI has increased the incidence of AKI to varying degrees [17,18,19,20]. We used two different UO criteria in our study and found that using different UO criteria also resulted in different incidences. More patients with AKI were identified using UOmean than using UOcont. Regarding calculation, UOcont was more stringent in terms of UO, which may be the reason that UOcont only identified a few patients with AKI. Some studies have suggested that UO should be considered a continuous physiological variable rather than a parameter over a certain time interval [21,22].

Regarding the difference in the incidence of AKI by stage, we found a higher incidence of stage 1 AKI in UOmean and UOcont, especially in UOmean, than that in SCR. We considered that UOmean may be highly sensitive in mild AKI identification. In stage 2 AKI, the incidence of UOcont-AKI and SCR-AKI was <1%, which is much lower than the incidence of UOmean-AKI. These results are consistent with the findings reported by Allen et al., who suggested that the use of UOmean does not only lead to overdiagnosis but also to misclassification of stage 2 AKI [16]. The UO criteria are better and more sensitive in identifying mild-to-moderate AKI [21,23,24]. We believe that the low incidence of moderate-to-severe UOcont-AKI may be because ICU physicians do not ignore persistent oliguria and intervene by immediate fluid resuscitation or use of diuretics to increase UO, making it difficult to identify patients by UOcont or classify them as having a more severe stage.

Our results showed differences in the length of ICU stay and hospital stay for patients with AKI compared with non-AKI patients under all three criteria. There is still disagreement regarding the association between UO criteria and long-term clinical outcomes. Some studies have shown that UO criteria are not associated with long-term mortality and are worse predictors of death than SCR criteria [25,26,27]. Additionally, there are opposing views, with some studies suggesting that UO criteria are more helpful in identifying patients with poor prognosis [28,29,30].

Moreover, the mechanisms underlying the association between oliguria and mortality remain unclear. Patients with oliguric AKI are likely to be in a state of fluid overload or fluid imbalance for various reasons [31], and such patients are more likely to receive fluid resuscitation in the clinical setting. This may also cause greater fluid overload in these patients, which is strongly associated with poor prognosis and early onset of complications [32]. Additionally, some studies pointed out that AKI in the ICU is a complex complication, and that hemodynamic instability, hypovolemia, and inflammation can lead to AKI and are strongly associated with the risk of death [33,34]. It is possible that AKI is not directly the true cause of death, so we need to further investigate the impact of oliguria on adverse clinical outcomes.

This study aimed to investigate whether the current methods of AKI identification, with different interpretations in the KDIGO definition, have an impact on the incidence and clinical outcome of AKI after non-cardiac surgery. This is because we found a general inconsistency between the use of SCR criteria and UO criteria in AKI-related studies; SCR criteria were used more often. We summarized a total of 120 clinical studies related to postoperative AKI using the KDIGO guidelines definition in recent years. Eighty-six studies used only SCR criteria. Eighteen studies explained the reasons for not using UO criteria, which included factors such as influence of diuretics on UO, inaccurate collection of UO, and other factors. Thirty-four studies used the full KDIGO guidelines to define AKI, but only six studies provided specific description of how to use the UO criteria. Therefore, we found that the current clinical studies related to postoperative AKI differed significantly in their definitions of AKI, and these differences may contribute to the occurrence of heterogeneous study results.

Our study has some limitations. First, it was a single-center retrospective study, which led to a limited number of patients, and it is unclear whether our results can be generalized at this point. Second, we did not consider the use of fluid balance and diuretics in the ICU. Excessive fluid balance may lead to problems with creatinine dilution, which may reduce the identification of AKI by SCR criteria, and the use of diuretics may alter UO, thereby affecting the identification of AKI by the UO criteria. Third, in our study, there was an overlap in the outcome population due to our study methodology, which led to our inability to confirm the veracity of the results obtained in this part of our study when we did the analysis related to clinical outcomes. The relatively disparate number of patients with AKI under each criterion and the fewer patients who died at the 90-day and 1-year after surgery may lead to statistical errors in our results. In addition, our results may be confounded by other factors that may affect mortality that are not known to us. Fourth, because the number of deaths with stage 2 and 3 AKI under each criterion in our study was too small, we were unable to verify the association between each severity of AKI and clinical outcomes. Fifth, because we did not exclude patients with ICU admissions of less than 48 h, this may have led to a decrease in our identification of patients with stage 2 and 3 AKI, as stage 2 and 3 AKI require a longer time window for diagnosis. In addition, as this was a retrospective study, we were unable to obtain renal function data at specific time points postoperatively, resulting in our inability to investigate and assess changes in renal function in patients postoperatively.

## 5. Conclusions

We found that the incidence and clinical outcomes of AKI after non-cardiac surgery varied considerably with the use of different criteria. We suggest that greater attention should be paid to the UO criteria; additionally, we recommend that further research should be carried out on the contribution of UO criteria to the identification and poor prognosis of AKI.

## Figures and Tables

**Table 1:** Patient characteristics.

	All Patients
Female sex, *n* (%)	284 (36.6)
Age, years, median (IQR)	68.8 (57.0–75.0)
BMI, kg/m^2^, median (IQR)	22.2 (20.0–24.7)
**Preoperative biochemical indicators, median (IQR)**	
Hemoglobin, g/dL	12.9 (11.5–14.0)
Albumin, g/dL	4.0 (3.7–4.3)
Serum creatinine, mg/dL	0.78 (0.64–0.93)
BUN, mg/dL	14.6 (11.8–18.0)
eGFR, mL/min/1.73 m^2^	70.6 (59.6–83.1)
**Comorbidities, *n* (%)**	
Hypertension	336 (43.2)
Diabetes	182 (23.4)
History of cancer	233 (30.0)
CKD (>G2)	200 (25.7)
**Information of surgery, *n* (%)**	
Abdominal surgery	338 (43.5)
Thoracic surgery	295 (38.0)
Other surgery	100 (12.9)
Emergency surgery	80 (10.3)
Laparoscopic surgery	361 (46.5)
**Information of anesthesia, *n* (%)**	
ASA-PS I	
	162 (20.9)
II	452 (58.2)
III	158 (20.3)
IV	5 (0.6)
TIVA	283 (36.4)
Anesthesia duration, h	6.3 (4.6–9.0)

BMI = body mass index; BUN = blood urea nitrogen; eGFR = estimated glomerular filtration rate; CKD = chronic kidney disease; ASA-PS = American Society of Anesthesiologists physical status; TIVA = total intravenous anesthesia; IQR = interquartile range.

**Table 2:** The association between AKI and non-AKI patients and clinical outcomes under different criteria.

	UOmean			UOcont			SCR		
	AKI (*n* = 257)	NO AKI (*n* = 520)	*p*	AKI (*n* = 61)	NO AKI (*n* = 716)	*p*	AKI (*n* = 16)	NO AKI (*n* = 761)	*p*
Length of ICU stay days, median (IQR)	2 (2–5)	2 (2–2)	<0.001	3 (2–5)	2 (2–3)	<0.001	4 (2–6)	2 (2–3)	0.001
Length of hospital stay days, median (IQR)	21 (14–31)	14 (10–24)	<0.001	24 (16–34)	16 (11–26)	<0.001	32 (26–44)	16 (11–26)	<0.001
90-day mortality, *n* (%)	8 (3.1)	2 (0.4)	0.002	1 (1.6)	9 (1.3)	0.799	1 (6.3)	9 (1.2)	0.075
1-year mortality, *n* (%)	30 (11.7)	36 (6.9)	0.025	8 (13.1)	58 (8.1)	0.178	3 (18.8)	63 (8.3)	0.137

ICU = intensive care unit; IQR = interquartile range.

## Data Availability

Data supporting the results of this study are available from the corresponding authors upon request.

## Supplementary Materials

The following supporting information can be downloaded at: https://www.mdpi.com/article/10.3390/jcm11195589/s1, Table S1: Results of univariate logistic regression and multivariate logistic regression of other factors with 1-year mortality, Table S2: Baseline characteristics of AKI and non-AKI patients by different KDIGO criteria, Table S3: Unadjusted and adjusted hazard ratios for AKI with 90-day mortality and 1-year mortality under different criteria using KDIGO guidelines.
