# Supplementary material for: Impact of Different KDIGO Criteria on Clinical Outcomes for Early Identification of Acute Kidney Injury after Non-Cardiac Surgery
Source: J Clin Med. 2022 Sep 23;11(19):5589. doi: 10.3390/jcm11195589 (PMC9571209; doi:10.3390/jcm11195589)
Supplement: Supplementary file 1 [file jcm-11-05589-s001.zip › jcm-1863593-Supplementary.pdf]

Supplementary Materials

Supplementary Table S1. Results of univariate logistic regression and multivariate logistic regression of other factors with 1-year mortality.

1-year mortality	Univariate logistic regression		Multivariate logistic regression	
	Odds Ratio (95%CI)	P value	Odds Ratio (95%CI)	P value
Sex	0.48 (0.27-0.88)	0.017	0.74 (0.39-1.43)	0.368
Age	1.01 (0.99-1.03)	0.371	-	
BMI	0.87 (0.81-0.94)	<0.001	0.91 (0.84-0.99)	0.023
Pre-Hemoglobin	0.77 (0.68-0.89)	<0.001	0.94 (0.78-1.13)	0.52
Pre-albumin	0.37 (0.24-0.55)	<0.001	0.56 (0.31-1.01)	0.055
Pre-BUN	1.03 (0.99-1.08)	0.097	-	
Pre-SCr	0.76 (0.28-2.09)	0.599	-	
Pre-eGFR	1.01 (0.99-1.02)	0.187	-	
Hypertension	1.44 (0.87-2.38)	0.158	-	
Diabetes	1.25 (0.71-2.21)	0.441	-	
History of cancer	0.87 (0.49-1.52)	0.615	-	
CKD	0.92 (0.51-1.65)	0.771	-	
Emergency surgery	2.40 (1.24-4.63)	0.009	2.51 (1.19-5.27)	0.015
Laparoscopic	0.78 (0.47-1.30)	0.345	-	
ASA>2	1.34 (0.75-2.40)	0.32	-	
TIVA	0.44 (0.24-0.81)	0.009	0.98 (0.49-1.96)	0.96
Anesthesia time	1.28 (1.17-1.39)	<0.001	1.26 (1.15-1.39)	<0.001

BMI = body mass index; BUN = blood urea nitrogen; eGFR = estimated glomerular filtration rate; CKD = chronic kidney disease; ASA-PS = American Society of Anesthesiologists physical status; TIVA = total intravenous anesthesia.

Supplementary Table S2. Baseline characteristics of AKI and non-AKI patients by different KDIGO criteria.

	UOmean			UOcont			SCR		
	AKI (N=257)	NO AKI (N=520)	p	AKI (N=61)	NO AKI (N=716)	p	AKI (N=16)	NO AKI (N=761)	p
Female sex, n (%)	77 (30.0)	207 (39.8)	0.007	18 (29.5)	266 (37.2)	0.23	5 (31.3)	279 (36.7)	0.66
Age, years, median (IQR)	68.6 (58.9-75.4)	68.8 (55.4-74.8)	0.63	68 (57.4-75.1)	68.8 (56.8-75.1)	0.53	70.6 (62.0-75.0)	68.7 (56.3-75.0)	0.44
BMI, kg/m², median (IQR)	22.8 (20.5-25.2)	22.1 (19.7-24.4)	0.007	23.5 (20.9-25.1)	22.2 (19.9-24.7)	0.12	21.7 (19.2-25.4)	22.2 (20-24.7)	0.98
**Preoperative biochemical indicators**, median (IQR)									
Hemoglobin, g/dL	12.6 (11.3-13.8)	13 (11.7-14.1)	0.04	12.2 (11.3-13.5)	12.9 (11.6-14.0)	0.03	12 (10.8-13.3)	12.9 (11.5-14.0)	0.09
Albumin, g/dL	4 (3.5-4.3)	4.1 (3.7-4.4)	<0.001	4 (3.5-4.3)	4.1 (3.7-4.4)	0.09	3.7 (3.2-4.1)	4.1 (3.7-4.3)	0.02
Serum creatinine, mg/dL	0.79 (0.65-0.90)	0.77 (0.64-0.94)	0.92	0.8 (0.66-0.88)	0.77 (0.64-0.93)	0.9	0.88 (0.76-1.12)	0.77 (0.64-0.92)	0.09
BUN, mg/dL	14.4 (11.3-17.6)	14.6 (12.1-18.2)	0.03	14.6 (11.8-17.4)	14.6 (11.8-18.0)	0.88	18 (1.32-21.4)	14.5 (11.8-18.0)	0.1
eGFR, mL/min/1.73m²	71.2 (60.9-84.1)	69.8 (58.5-82.8)	0.12	70.7 (60.9-84.2)	70.6 (58.3-83.1)	0.64	59.2 (45.2-74.8)	70.7 (69.9-83.1)	0.05
**Comorbidities**, n (%)									
Hypertension	124 (48.3)	212 (40.8)	0.048	26 (42.6)	310 (43.3)	0.92	11 (68.8)	325 (42.7)	0.037
Diabetes	64 (24.9)	118 (22.7)	0.49	19 (31.2)	163 (22.8)	0.14	5 (31.3)	177 (23.3)	0.46
History of cancer	62 (24.1)	171 (32.9)	0.012	14 (23.0)	219 (60.6)	0.21	3 (18.8)	230 (30.2)	0.32
CKD(>G2)	54 (21.0)	146 (28.1)	0.034	13 (21.3)	187 (26.1)	0.41	8 (50.0)	192 (25.2)	0.025
Information of surgery, n (%)			<0.001			<0.001			0.005
Abdominal surgery	149 (58.0)	191 (36.7)		41 (67.2)	299 (41.8)		14 (87.5)	326 (42.9)	

Thoracic surgery	66 (25.7)	229 (44.0)		8 (13.1)	287 (40.1)		1 (6.3)	294 (38.6)	
Other surgery	42 (16.3)	100 (19.2)		12 (19.7)	130 (18.2)		1 (6.3)	141 (18.5)	

Supplementary Table S2. *Cont.*

	UOmean			UOcont			SCR		
	AKI (N=257)	NO AKI (N=520)	p	AKI (N=61)	NO AKI (N=716)	p	AKI (N=16)	NO AKI (N=761)	p
Emergency surgery	32 (12.5)	47 (9.0)	0.14	4 (6.6)	75 (10.5)	0.33	2 (12.5)	77 (10.1)	0.76
Laparoscopic surgery	95 (37.0)	266 (51.2)	<0.001	19 (31.2)	342 (47.8)	0.012	4 (25.0)	357 (47.0)	0.08
Information of anesthesia, n (%)									
ASA-PS			0.27			0.36			0.1
I	51 (19.8)	111 (21.4)		17 (27.9)	145 (20.3)		1 (6.3)	161 (21.2)	
II	143 (55.6)	309 (59.4)		42 (68.9)	421 (58.8)		8 (50.0)	444 (58.3)	
III	60 (23.4)	98 (18.9)		23 (37.7)	146 (20.4)		7 (43.8)	151 (19.8)	
IV	3 (1.2)	2 (0.4)		1 (1.6)	4 (0.6)		0 (0.0)	5 (0.7)	
TIVA	69 (26.9)	214 (41.2)	<0.001	13 (21.3)	270 (37.7)	0.011	0 (0.0)	283 (37.2)	0.002
Anesthesia duration, h	7.6 (5.3-10.1)	5.7 (4.4-8.2)	<0.001	8.5 (7.0-10.9)	6 (4.6-8.8)	<0.001	10.2 (8.1-9.0)	6.2 (4.6-9.0)	<0.001

BMI = body mass index; BUN = blood urea nitrogen; eGFR = estimated glomerular filtration rate; CKD = chronic kidney disease; ASA-PS = American Society of Anesthesiologists physical status; TIVA = total intravenous anesthesia.

Supplementary Table S3. Unadjusted and adjusted hazard ratios for AKI with 90-day mortality and 1-year mortality under different criteria using KDIGO guidelines.

	90-day Mortality	1-year Mortality
**UOmean-AKI**		
Unadjusted hazard ratio (95% CI)	8.18 (1.74-38.54)	2.56 (1.37-4.78)
Adjusted hazard ratio (95% CI)	5.72(1.15-28.59)	1.72 (0.89-3.30)
**UOcont-AKI**		
Unadjusted hazard ratio (95% CI)	1.30 (0.16-10.29)	1.72 (0.68-4.40)
Adjusted hazard ratio (95% CI)	0.83 (0.10- 6.85)	1.09 (0.42- 2.85)
**SCR-AKI**		
Unadjusted hazard ratio (95% CI)	5.32 (0.67-42.02)	4.25 (1.31-13.79)
Adjusted hazard ratio (95% CI)	2.90 (0.33-25.56)	1.93 (0.57- 6.59)
